# Eryptosis and Malaria: New Experimental Guidelines and Re-Evaluation of the Antimalarial Potential of Eryptosis Inducers

**DOI:** 10.3389/fcimb.2021.630812

**Published:** 2021-03-12

**Authors:** Coralie Boulet, Taylah L. Gaynor, Teresa G. Carvalho

**Affiliations:** Department of Physiology, Anatomy and Microbiology, School of Life Sciences, La Trobe University, Melbourne, VIC, Australia

**Keywords:** eryptosis, *Plasmodium*, malaria, cell death, eryptosis inducers, phosphatidylserine exposure, erythrocyte (human)

## Abstract

Erythrocytes possess an unusual programmed cell death mechanism termed eryptosis, and several compounds have been previously claimed to induce eryptosis *in vitro*. Malaria parasites (genus *Plasmodium*) reside in erythrocytes during the pathogenic part of their life cycle, and the potential of several eryptosis inducers to act as antimalarials has been tested in recent years. However, the eryptosis-inducing capacity of these compounds varies significantly between eryptosis-focused studies and malaria investigations. Here, we investigated the reasons for these discrepancies, we developed a protocol to investigate eryptosis in malaria cultures and we re-evaluated the potential of eryptosis inducers as antimalarials. First, we showed that eryptosis read-out *in vitro* is dependent on culture conditions. Indeed, conditions that have consistently been used to study eryptosis do not support *P. falciparum* growth and prime erythrocytes for eryptosis. Next, we defined culture conditions that allow the detection of eryptosis while supporting *P. falciparum* survival. Finally, we selected six eryptosis-inducers based on their clinical use, molecular target and antimalarial activities, and re-evaluated their eryptosis inducing capacities and their potential as antimalarials. We demonstrate that none of these compounds affect the viability of naïve or *P. falciparum*-infected erythrocytes *in vitro*. Nevertheless, three of these compounds impair parasite development, although through a mechanism unrelated to eryptosis and yet to be elucidated. We conclude that careful consideration of experimental set up is key for the accurate assessment of the eryptosis-inducing potential of compounds and their evaluation as potential antimalarials.

## Introduction

Malaria is a devastating infectious disease that considerably affects the lives of millions worldwide. Despite a recent global decrease in malaria incidence, this progress is reaching a plateau, with 229 million malaria cases in 2019 (World Health Organization, 2020). Challenges, such as the lack of a long-lasting efficient vaccine and the spread of antimalarial drug resistance, represent a serious threat to malaria elimination (White, 2008; World Health Organization, 2016). Therefore, novel alternative treatment options are urgently needed. Infection of erythrocytes by *Plasmodium* spp. parasites leads to the development of all symptoms associated with malaria, hence most antimalarials developed to date target the parasite in its blood stages. However, parasites quickly develop resistance against these antimalarials. For this reason, host-directed therapies have emerged in recent years as an attractive and novel strategy to tackle malaria infections (Glennon et al., 2018), combining a reduced likelihood for the emergence of drug resistance and drug repurposing opportunities. Many intracellular pathogens, including the *Plasmodium* liver stages (van de Sand et al., 2005), have been shown to inhibit or delay host cell death pathways to promote their own survival. As such, host cell death factors appear as ideal targets for antipathogen therapeutics (Lamkanfi and Dixit, 2010). Further, compounds that inhibit human cell death factors are available, many of which were developed for cancer therapies, and could be repurposed in the development of new host-directed therapeutics for malaria.

Erythrocytes display a form of programmed cell death distinct from senescence termed eryptosis (Bratosin et al., 2001). Eryptosis hallmarks closely resemble those of apoptosis and include i) an increase in intracellular calcium levels, ii) phosphatidylserine (PS) exposure to the cell surface (leading to erythrocyte clearance by macrophages), iii) cell shrinkage, and iv) membrane blebbing (Bratosin et al., 2001; Lang et al., 2005). The potential of eryptosis-inducing compounds in antimalarial drug discovery has drawn attention in recent years (Bobbala et al., 2010a; Fallatah and Georges, 2017). A dozen eryptosis inducers have been tested on *Plasmodium* parasites, and most of the compounds tested were reported to affect parasite viability *in vitro* and in murine models (reviewed in Boulet et al., 2018). In these studies, the eryptosis inducers induced PS exposure of infected erythrocytes. However, none of the compounds tested *in vivo*, and few of those tested *in vitro*, induced PS exposure of uninfected erythrocytes (reviewed in Boulet et al., 2018). These observations contrast with original reports of the eryptosis inducing activity of such compounds on naïve erythrocytes. The current discrepancies emerging from the literature about the ability (or inability) of eryptosis inducers to induce erythrocyte death might stem from significant differences in the culture conditions used in the assays. Indeed, eryptosis-focused studies maintain erythrocytes in Ringer solution (a basic saline buffer), at 0.4% hematocrit—conditions that would not support *Plasmodium* growth (Lang et al., 2011; Bigdelou and Farnoud, 2020). On the other hand, *Plasmodium in vitro* cultures are conducted in RPMI 1640 media complemented with Albumax (a serum replacement) at 4% hematocrit (Trager and Jensen, 1976). The contribution of these differences in culture conditions has never been tested on eryptosis read-out and is rarely discussed in studies investigating eryptosis inducers in a malaria context (Bobbala et al., 2009; Alesutan et al., 2010; Bobbala et al., 2010b; Siraskar et al., 2010). Protein-containing media used in *Plasmodium* cultures has been hypothesized to contribute to the absence of PS exposure induction, through sequestration of the eryptosis-inducing compound (Bobbala et al., 2010a), and human plasma has been suggested to contain pro-survival factors protecting against eryptosis induction (Walsh et al., 2002). However, these hypotheses remain to be tested. More recently, two step-by-step protocols have been published to improve reproducibility of eryptosis experiments (Jemaà et al., 2017; Bigdelou and Farnoud, 2020). Noteworthy, these protocols have been conducted in Ringer solution at 0.4% hematocrit, conditions that are not suitable for *Plasmodium* growth. Therefore, the contribution of culture conditions has yet to be investigated in the context of eryptosis of naïve and *Plasmodium*-infected erythrocytes, and such is the aim of the current study.

In an attempt to address current major discrepancies emerging from the eryptosis literature, we investigated why eryptosis inducers do not induce eryptosis in malaria studies. Consequently, we provided guidelines to measure eryptosis in *P. falciparum*-infected red blood cells, and finally we re- evaluated the potential of eryptosis inducers as antimalarial candidates. When comparing cell culture methodologies used for uninfected erythrocytes and for erythrocytes infected with *P. falciparum*, the most virulent human malaria parasite, we identified that Albumax supplementation strongly protects against PS exposure and hemolysis. The media itself (Ringer or RPMI) and the hematocrit (0.4% or 4%) also influence eryptosis hallmarks. Based on our observations, we provide guidelines for future investigations of eryptosis in malaria *in vitro* studies. Further, using these new protocol guidelines we demonstrate that none of the eryptosis inducers tested in this study induce eryptosis of naïve or *P. falciparum*-infected erythrocytes, although three compounds impair *P. falciparum* growth. Overall, the present study unveils reasons behind discrepancies observed between eryptosis-focused studies and those conducted in the context of malaria infections. Moreover, we have shown that “eryptosis inducers” exert their antiparasitic effects independently of eryptosis and *via* a mechanism yet to be unveiled. We explore and discuss the potential of the compounds tested in this study as antimalarials, considering their current and potential use in the clinic.

## Methods

### *In Vitro* Cell Culture

Use of human erythrocytes was approved by the La Trobe University Research Ethics Committee (ethics number HEC17‐013) and an Australian Red Cross Blood Service Agreement (Deed 19-05VIC-01). Red blood cell populations are intrinsically heterogeneous and variable between donors, therefore each experiment presented in this study was conducted a minimum of three independent times, each with a different batch of blood. Human red cells were processed as soon as possible upon collection to minimize erythrocyte aging. Naïve (non-infected) erythrocytes and *Plasmodium falciparum* 3D7-infected erythrocytes were cultured at 37°C, in low oxygen conditions (1% O_2_, 5% CO_2_, 94% N_2_), at 4% or 0.4% hematocrit, in incomplete RPMI media (iRPMI: RPMI 1640 HEPES, 50 mg/L hypoxanthine, 10 mg/L gentamicin, pH 6.74), complete RPMI media (cRPMI: incomplete RPMI supplemented with 2.25 g/L sodium bicarbonate and 5 g/L AlbuMAX II), or Ringer solution (1mM CaCl_2_, 5mM glucose, 32 mM HEPES pH 7.4, 5 mM KCl, 1mM MgSO4, 125mM NaCl). *P. falciparum* standard *in vitro* culture conditions were conducted as previously described (Trager and Jensen, 1976).

### Eryptosis Assay and Flow Cytometry Analysis

Previously described eryptosis inducers were selected for this study based on their use (or potential use) in the clinic, their known targets and/or their previously described antimalarial activity (**Table 1**): amiodarone HCl (Selleck Chem, #S1979), apigenin (Selleck Chem, #S2262), BAY 43-9006 (AdipoGen Life Sciences, #AG-CR1-0025), benzethonium chloride (Selleck Chem, #S4162), cordycepin (Cayman Chemicals, #14426), ionomycin (ThermoFisher, #I24222) and oridonin (Selleck Chem, #S2335). Drug assays measuring eryptosis were carried out in 100 μl or 500 μl of culture when using 4% or 0.4% hematocrit conditions, respectively. DMSO, the vehicle, was used for the no-drug conditions. Cells were incubated at 37°C, in low oxygen conditions for 1 h to 48 h depending on the assay. Each experiment was performed in triplicate unless otherwise indicated and cells were analyzed by flow cytometry. Cells were washed in PBS, resuspended in Thiazole Orange (BD Retic-Count™, BD Bioscience) containing 4 μM Hoechst 33342 (Invitrogen) for parasite RNA and DNA detection respectively (Grimberg, 2011), and incubated in the dark for 40 min at 37°C. Cells were washed in staining buffer (5 mM CaCl_2_, 5 mM glucose, 32 mM HEPES pH7.4, 5 mM KCl, 1 mM MgSO4, 125 mM NaCl), resuspended in 0.3 μM of intracellular calcium dye x-Rhod-1 AM (Invitrogen) and incubated in the dark at 37°C for 30 min. Subsequently, cells were washed in staining buffer and incubated with Annexin V-PE (1:20 dilution in staining buffer; BD Bioscience) at 37°C for 20 min in the dark for the staining of surface-exposed phosphatidylserine. Cells were resuspended in 200 μl staining buffer and analyzed within the hour using a CytoFLEX S flow cytometer (Beckman Coulter). 100,000 total events were recorded for each condition at a flow rate of 10 μl/min. Data were analyzed using FlowJo V10. For PS exposure measure, gating was set using data obtained with naïve erythrocytes DMSO control. Infected cells were gated using Hoechst fluorescence. The percentage of Annexin V-PE positive cells, the mean forward scatter height (FSC-H) and mean x-Rhod-1 fluorescence area (PE-A) were acquired for naïve and infected-erythrocytes.

**Table 1:** Six previously published eryptosis compounds have been selected to be tested in this study, based on their current or potential clinical use and their known molecular targets. Further, two compounds have been previously tested on malaria parasites.

Compound	Molecular target	Clinical use or potential use	Published eryptosis induction concentration	Effect on *P. falciparum/P. berghei*
**Clinically approved**				
**Amiodarone**	Calcium, potassium and sodium channels inhibitor	Anti-arrhythmic agent (Zimetbaum, 2012; National Center for Biotechnology Information, 2020a)	5 µM, (Nicolay et al., 2007)	Reduced parasitemia (Pf, Pb) and increased mice survival (Pb) (Bobbala et al., 2010b)
**BAY 43-9006** (Sorafenib, Nexavar)	Raf kinase inhibitor	Anti-cancer (hepatocellular carcinoma) (Ng and Chen, 2006)	1 µM, (Lupescu et al., 2012)	–
**Clinical trials**				
**Cordycepin**	AMPK activator	Isolated from the fungus *Cordyceps militaris* Clinical trials I/II: Anti-cancer capacities (Wang et al., 2019)	31 µM, (Lui et al., 2007)	–
**Herbal medicine compounds**				
**Apigenin**	Kinases inhibitor	Extracted from chamomile Anti-inflammatory, anti-cancer capacities (National Center for Biotechnology Information, 2020b)	15 µM, (Zbidah et al., 2012)	Decreased parasitemia (Pb) (Amiri et al., 2018)
**Oridonin**	Not well understood	Extracted from *Isodon rubescens* Anti-inflammatory, anti-cancer capacities (Ding et al., 2016; Xu et al., 2018)	25 µM, (Jilani et al., 2011)	–
**Other**				
**Benzethonium**	Disrupts lipid bilayer (cationic detergent)	FDA approved as a skin disinfectant and preservative in vaccines (Geier et al., 2010; National Center for Biotechnology Information, 2020c) Anti-cancer capacities (Yip et al., 2006)	5 µM, (Lang et al., 2011)	–

### Hemolysis Assay

Following drug incubation, cells were spun down at 200 g for 5 min, the supernatant was collected, and absorbance was read at 405 nm on a CLARIOstar^®^ microplate reader. Absorbance of culture media alone was considered as baseline.

### Parasite Growth Inhibition Assay

Compound-induced parasite growth inhibition was measured as previously described (Morahan et al., 2020). Briefly, an asynchronous parasite culture at 0.25% parasitemia and 2% hematocrit was incubated in a 96-well plate with a two-fold serial dilution of compound over 72 h in standard culture conditions (cRPMI, 37°C, low oxygen). Compounds (at 10 mM in DMSO) were added to the first well of the dilution series to produce a 50 µM drug concentration (and 0.5% (v/v) DMSO) and diluted two-fold until a concentration of 0.19 nM compound was reached. Each condition was conducted in triplicates, subject to a freeze/thaw cycle and incubated with the DNA intercalant SYBR Gold following manufacturer instructions (SYBR™ Gold Nucleic Acid Stain, Invitrogen). The average fluorescence between triplicates was measured at 495 nm using a CLARIOstar^®^ microplate reader and analyzed as a proxy of parasite replication. Compound-induced parasite growth inhibition was normalized to a no-drug control [0.5% (v/v) DMSO] and to a parasite growth inhibition positive control [50 µM artemisinin (ART) and 50 µM chloroquine (CQ) in 0.5% (v/v) DMSO], using the following formula: (ART CQ with (ART + &CQ) or (ART &CQ) ∗ 100. Growth inhibition was plotted against the logarithm of drug concentration using GraphPad Prism v8. The resulting non-linear regression curve determines the compound concentration (µM) that inhibits growth of 50% of the parasite population (IC_50_). For each compound five independent biological replicates were performed.

### Statistical Analysis

Data is expressed as the mean +/- standard deviation (SD). One-way ANOVA with Tukey’s multiple comparisons test was performed to calculate the *p* value (*p* > 0.05: ns; *p ≤* 0.05: *; *p ≤* 0.01: **; *p ≤* 0.001: ***; *p ≤* 0.0001: ****) using GraphPad Prism v8.

## Results

### Establishing Guidelines for the Detection of Eryptosis

To study eryptosis of *P. falciparum*-infected erythrocytes we started by testing the previously described eryptosis inducer BAY 43-9006 (Lupescu et al., 2012). We observed that BAY 43-9006 induced PS exposure in Ringer solution, but not when tested in culture conditions used to grow *P. falciparum* (*i.e*. in cRPMI and 4% hematocrit). This prompted us to explore the impact of cell culture media (Ringer solution *vs*. RPMI), hematocrit (0.4% *vs*. 4%) and role of Albumax on the baseline eryptosis level of non-infected erythrocytes. We developed guidelines to study eryptosis in the context of malaria and tested if the strong eryptosis inducer ionomycin (Bigdelou and Farnoud, 2020) was a suitable positive control for our eryptosis assays.

#### BAY 43-9006 Induces Eryptosis Exclusively in Ringer Solution

**Figure 1:**
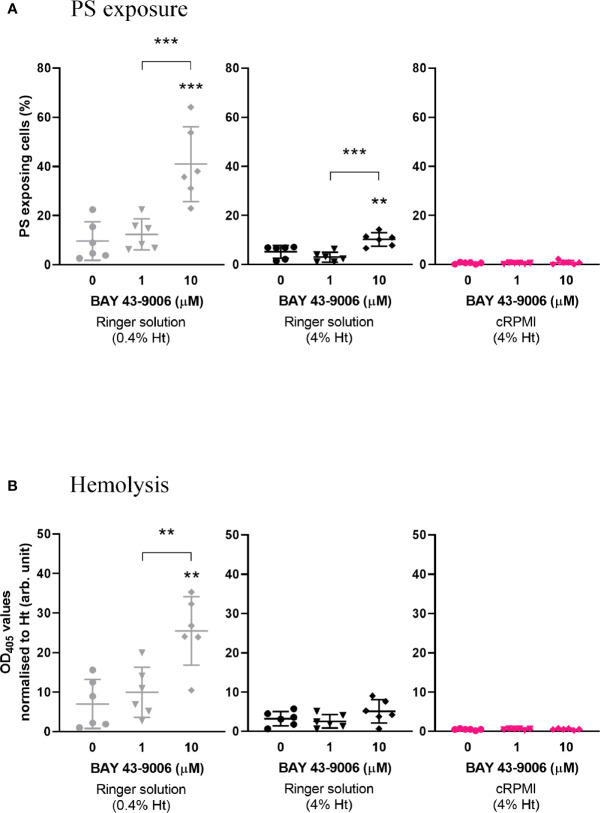
The previously described eryptosis inducer BAY 43-9006 only induces an increase of PS exposure and hemolysis of naïve erythrocytes in Ringer solution. Percentage of PS-exposing cells **(A)** and hemolysis (normalized by hematocrit) **(B)** of naïve erythrocytes cultured for 48 h in the presence of 0, 1, or 10 µM BAY 43-9006 in Ringer solution at 0.4% or 4% hematocrit, or in malaria complete RPMI (cRPMI) at 4% hematocrit. The data presented here corresponds to six independent experiments performed in triplicate (N=6). Individual data points represent the means of the three technical replicates for each experiment. The bars represent the mean and SD of the six independent experiments. Unless indicated otherwise, compare to the first condition. *p* > 0.05: ns; *p ≤* 0.05: *; p ≤ 0.01: **; p ≤ 0.001: ***; p ≤ 0.0001: ****.

We sought to investigate the effect of the published eryptosis inducer BAY 43-9006 (Lupescu et al., 2012), a Raf kinase inhibitor and approved anti-cancer drug. Prior to testing this compound on parasites, we began by investigating whether BAY 43-9006 would induce eryptosis of non-infected erythrocytes in malaria culture conditions. Erythrocytes were exposed to 0, 1 and 10µM of BAY 43-9006 for 48 h in Ringer solution (either at 0.4% hematocrit, used to describe BAY 43-9006 as an eryptosis inducer (Lupescu et al., 2012), or at 4% hematocrit, routinely used to culture *P. falciparum*), or in cRPMI (4% hematocrit) (**Figure 1**). As anticipated, BAY 43-9006 significantly increased the percentage of PS-exposing cells at 10µM (from 10% to 41%; *p*=0.0003) in Ringer solution (0.4% hematocrit) when compared to the no-drug control. When adjusting the hematocrit to 4%, the increase in PS-exposing cells was still observed, albeit to a lesser extent (from 5% to 10% of PS-exposing cells, *p*=0.0083). However, in cRPMI in the presence of 10µM of BAY 43-9006, no significant increase in PS exposure was observed. Similarly, 10µM of BAY 43-9006 significantly induced hemolysis exclusively in Ringer at 0.4% hematocrit (*p*=0.0012), but not in Ringer at 4% hematocrit or in cRPMI at 4% hematocrit. These results demonstrate that BAY 43-9006 increases PS exposure and hemolysis levels under the culture conditions typically used for eryptosis studies, but not under conditions used for *P. falciparum* culture.

#### Culturing in Ringer Solution at Low Hematocrit Increases the Baseline Levels of PS Exposure and Hemolysis in Erythrocytes

**Figure 2:**
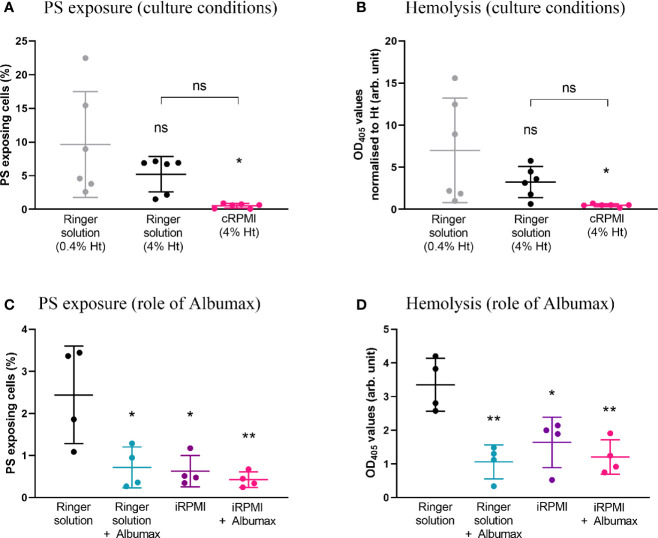
PS exposure and hemolysis levels of naïve erythrocytes strongly depend on cell culture parameters. Percentage of PS-exposing cells **(A)** and hemolysis (normalized by hematocrit) **(B)** of erythrocytes cultured for 48 h in either Ringer solution at 0.4% or 4% hematocrit, or in cRPMI at 4% hematocrit. The data presented corresponds to six independent experiments performed in triplicate (N=6). Percentage of PS-exposing cells **(C)** and hemolysis levels **(D)** of erythrocytes cultured for 48 h in either Ringer solution or incomplete RPMI (iRPMI) supplemented or not with Albumax (all conditions at 4% hematocrit). The data presented corresponds to four independent experiments performed in triplicate (N=4). Individual data points represent the means of the three technical replicates for each experiment. The bars represent the mean and SD of the four to six independent experiments. Unless indicated otherwise, compare to the first condition. *p* > 0.05: ns; *p ≤* 0.05: *; p ≤ 0.01: **; p ≤ 0.001: ***; p ≤ 0.0001: ****.

To test the influence of specific culture conditions on baseline levels of eryptosis, and in the absence of any drug, naïve erythrocytes were incubated in either Ringer solution (at 0.4 or 4% hematocrit) or in cRPMI at 4% hematocrit (**Figures 2A, B**). Following 48 h cell incubation, PS exposure and hemolysis were measured. A significantly higher level of PS exposure was observed in Ringer at 0.4% hematocrit when compared to cRPMI at 4% hematocrit (*p*=0.0127). In Ringer solution, when the hematocrit was adjusted to 4%, PS exposure was not significantly different, although it was around half the level of PS exposure seen in the 0.4% hematocrit condition. At 4% hematocrit, PS exposure was 10-times higher in Ringer than in cRPMI, however the difference was not significant either. Similar to PS exposure, hemolysis (normalized to hematocrit) was significantly higher in Ringer at 0.4% hematocrit compared to cRPMI at 4% hematocrit (*p*=0.0219). In Ringer solution, although hemolysis was halved at 4% hematocrit when compared to 0.4% hematocrit, the difference was not significant. After adjusting for hematocrit, no difference in hemolysis was observed between Ringer and cRPMI media, although once again, it was nearly 10-times higher in Ringer.

To compare the impact of the type of media alone, erythrocytes were incubated in Ringer solution or incomplete RPMI (iRPMI; the contribution of Albumax supplementation is assessed below) at 4% hematocrit. Note that 4% hematocrit was chosen for all subsequent experiments as it supports *P. falciparum* growth. PS exposure and hemolysis were measured after 48 h (**Figures 2C, D**). In Ringer solution, ~four-times more cells exposed PS (*p*=0.0103) and ~two-times more hemolysis was observed (p=0.0134) when compared to the iRPMI condition. This reveals that Ringer solution primes erythrocytes for eryptosis and/or that iRPMI protects erythrocytes from eryptosis.

#### Albumax Strongly Protects Erythrocytes From Increased PS Exposure and Hemolysis

Albumax (a serum replacement) is a component used for successful prolonged *Plasmodium in vitro* culture but is not used in eryptosis-focused studies. To determine whether Albumax was a contributing factor for the difference in PS exposure and hemolysis observed between culture conditions, we incubated erythrocytes in Ringer solution +/- Albumax or in incomplete RPMI (iRPMI) +/- Albumax for 48 h at 4% hematocrit (**Figures 2C, D**). Compared to Ringer solution alone, addition of Albumax to Ringer solution significantly reduced both PS exposure (*p*=0.0143) and hemolysis levels (*p*=0.0016) by ~3-fold. Similarly, compared to iRPMI alone, addition of Albumax to iRPMI decreased PS exposure and hemolysis levels, though not significantly. We conclude that the addition of Albumax protects erythrocytes from increased PS exposure and hemolysis.

#### Ionomycin Induces PS Exposure in All Conditions Tested

**Figure 3:**
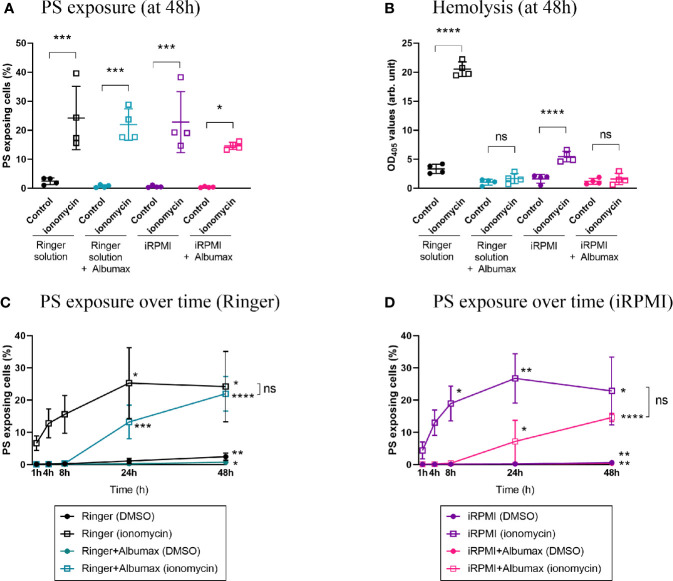
Ionomycin induces PS exposure in all conditions but Albumax delays eryptosis. Erythrocytes were cultured in Ringer (+/- Albumax) or incomplete RPMI (+/- Albumax) in the presence of the calcium ionophore ionomycin (1 µM) or carrier control (DMSO). PS exposure **(A)** and hemolysis **(B)** levels were measured after 48 h. Further, PS levels were determined after 1, 4, 8, 24, and 48 h of incubation in Ringer **(C)** or iRPMI **(D)**. The data presented corresponds to four independent experiments performed in triplicate (N=4). Individual data points represent the means of the three technical replicates for each experiment. The bars represent the mean and SD of the four independent experiments. *p* > 0.05: ns; *p ≤* 0.05: *; p ≤ 0.01: **; p ≤ 0.001: ***; p ≤ 0.0001: ****. For **(C, D)**: unless indicated otherwise, compare each timepoint to T_1h_ within each condition; only significant differences are indicated.

After establishing the baseline eryptosis levels in different culture conditions, we needed to validate an eryptosis inducer we could use as a positive control for subsequent experiments. To this effect we tested the well-known calcium ionophore and previously described eryptosis inducer ionomycin (Bratosin et al., 2001; Totino et al., 2011; Bigdelou and Farnoud, 2020). Erythrocytes were cultured in either Ringer (+/- Albumax) or iRPMI (+/- Albumax), in the presence of 1 μM ionomycin, at 4% hematocrit for 48 h (**Figures 3A, B**). Ionomycin significantly increased PS exposure in all conditions when compared to the DMSO control: Ringer (2.4% to 24%, *p*=0.0004), Ringer+Albumax (0.72% to 22%, *p*=0.0005), iRPMI (0.63% to 23%, *p*=0.0003) and iRPMI+Albumax (0.43% to 15%, *p*=0.0329). Similarly, hemolysis increased significantly in the presence of ionomycin, both in Ringer and iRPMI (p<0.0001). However, addition of Albumax to either media was sufficient to abolish ionomycin-induced hemolysis. Ionomycin-induced hemolysis in iRPMI was less pronounced than in Ringer (OD_405_ = 5.5 *vs.* 20.5, respectively, *p*<0.0001). Taken together, these results suggest that Ringer solution sensitizes erythrocytes to hemolysis, and that Albumax protects erythrocytes from ionomycin-induced lysis. Moreover, given that ionomycin induces PS exposure in all conditions, it emerged as an effective positive control to test eryptosis levels *in vitro*.

#### The Induction of PS Exposure Is a Fast Process and Is Delayed by Albumax

Eryptosis studies conducted to date have measured the cellular response to eryptosis stimulants following a 48 h incubation period (Jilani et al., 2011; Lang et al., 2011; Lupescu et al., 2012). Considering that PS exposure of apoptotic nucleated cells can be detected within a few hours (Bevers et al., 1983), we hypothesized that eryptosis responses might be a similarly fast process. To investigate the dynamics of PS exposure stimulated by ionomycin, erythrocytes were incubated with 1 μM ionomycin and PS exposure was measured between 1 and 48 h (**Figures 3C, D**). First, in the absence of Albumax and drug, PS exposure of erythrocytes slowly increased over time, both in Ringer solution (24-fold increase after 48 h, *p*=0.0011) and in iRPMI (6.4-fold increase after 48 h, *p*=0.0068). The dynamic of PS exposure was much faster in response to ionomycin. Indeed, 1 h after the addition of ionomycin, a strong increase in PS exposure was observed both in Ringer solution (62-fold increase compared to the no-drug condition, *p*<0.0001) and in iRPMI (45-fold increase, *p*=0.0032).

Compared to erythrocytes cultured in Ringer solution alone (T_1h_=0.1% and T_48h_=2.4%; *p*=0.0011), the increase in baseline PS exposure was significantly dampened by the addition of Albumax (T_1h_=0.1% and T_48h_=0.7%; *p*=0.0212) (**Figure 3C**). When ionomycin was added to erythrocytes, the increase in PS exposure was more pronounced in the no-Albumax condition. The increase was significant after only 1 h incubation in Ringer alone (T_1h_=0.1% in Ringer DMSO *vs*. 6.6% in Ringer ionomycin; *p*<0.0001), but was significant after 24 h in Ringer+Albumax (T_24h_=0.3% DMSO *vs*. 13.2% ionomycin; *p*=0.0476).

When cells were incubated in iRPMI (+/- Albumax), similar observations were made (**Figure 3D**). Baseline PS exposure increased modestly but significantly over time in both iRPMI alone (T_1h_=0.1% and T_48h_=0.6%; *p*=0.0068) and iRPMI+Albumax (T_1h_=0.1% and T_48h_=0.4%; *p*=0.0084). The addition of ionomycin to iRPMI alone induced a rapid and significant increase of PS exposure after only 1 h incubation (T_1h_=0.1% in DMSO *vs*. 4.4% in ionomycin; *p*=0.0032). In iRPMI+Albumax, however, ionomycin only induced a significant increase in PS exposure after 48 h (T_48h_=0.4% DMSO *vs*. 14.6% ionomycin; *p*=0.0120). Overall, Albumax appears to protect erythrocytes from PS exposure and to delay ionomycin-induced eryptosis.

#### Guidelines to Study Eryptosis in *P. falciparum In Vitro *Cultures

Our experimental data thus far demonstrated that testing the impact of eryptosis inducers on the viability of *P. falciparum*-infected erythrocytes requires conditions that allow the detection of any changes in eryptosis while supporting the survival of *P. falciparum*. Henceforth we systematically measured eryptosis phenotypes of cells cultured in iRPMI (without Albumax) at 4% hematocrit and exposed to a 4 h drug incubation period. A 4 h incubation period provides sufficient time for eryptotic changes to occur (*e.g.* we have demonstrated that PS exposure occurs within 1 h in the presence of ionomycin), and, based on our observations, is the maximum amount of time *P. falciparum* remains viable when cultured in iRPMI. Further, a short incubation time (4 h instead of 48 h) allows investigation of the impact of eryptosis inducers on specific *P. falciparum* stages (ring, trophozoite, schizont). Finally, we have validated ionomycin (1 μM) as a positive control of eryptosis under these conditions.

### Testing the Effect of Eryptosis Inducers on *P. falciparum* Cultures

After establishing new guidelines for measuring eryptosis levels of malaria parasite cultures and using ionomycin as a positive control, we re-evaluated the effect of selected eryptosis inducers on the viability of naïve and *P. falciparum*-infected erythrocytes within *in vitro* cultures.

#### Selection of Six Eryptosis Inducers of Interest

Six eryptosis inducers were selected based on their clinical use (or potential use), their known molecular targets and/or their demonstrated impact on *Plasmodium* development (summarized in **Table 1**).

Amiodarone is a clinically approved antiarrhythmic agent, active by inhibiting Na^+^/K^+^-ATPase and multiple ions channels, including Ca^2+^ channels (Zimetbaum, 2012; National Center for Biotechnology Information). Amiodarone was previously shown to induce an increase in PS exposure and cytosolic Ca^2+^ concentrations (Nicolay et al., 2007), and to impair *P. falciparum* and *P. berghei* growth (Bobbala et al., 2010b).

BAY 43-9006 (also called Sorafenib and Nexavar) is an approved anticancer drug that targets the Raf kinase (Ng and Chen, 2006). BAY 43-9006 was previously shown to induce a decrease in erythrocyte cell size, an increase in intracellular Ca^2+^ levels, PS exposure and hemolysis (Lupescu et al., 2012).

Cordycepin is an antibiotic (a nucleoside analogue) isolated from the fungus *Cordyceps sinensis* which is used in traditional Chinese medicine and sold as health food. Cordycepin is currently in clinical trials (phase I/II) to treat leukemia (National Cancer Institute, 2011), and was found to activate AMPK (Wang et al., 2019). Cordycepin was demonstrated to induce PS exposure and increased intracellular Ca^2+^ levels in mouse erythrocytes (Lui et al., 2007).

Apigenin is a key molecule from the traditional herbal remedy chamomile, which exhibits anti-inflammatory and anticancer activities through regulation of many signaling pathways, in particular through inhibition of various protein kinases (National Center for Biotechnology Information). Apigenin was shown to significantly decrease erythrocyte cell size, increase cytosolic Ca^2+^ concentrations and PS exposure (Zbidah et al., 2012), and to display antimalarial activity in mice (against *P. berghei*) (Amiri et al., 2018).

Oridonin is a molecule extracted from *Isodon* plants, traditionally used in Chinese medicine as anticancer and anti-inflammatory remedies (Ding et al., 2016). Oridonin and its derivates seem to induce apoptosis through a wide range of signaling pathways, which include p38, Jnk, Erk, ROS and FasL (Ding et al., 2016; Xu et al., 2018). In particular, one of oridonin derivate (HAO472) is currently in phase I clinical trials in China for the treatment of a type of leukemia (Ding et al., 2016). Oridonin has previously been shown to decrease erythrocytes cell size, to increase PS exposure, cytosolic Ca^2+^ concentrations, and hemolysis (Jilani et al., 2011).

Benzethonium chloride is a synthetic quaternary ammonium salt with antimicrobial properties, widely used in liquid antiseptics, in cosmetics (National Center for Biotechnology Information), and as a preservative in vaccines (Geier et al., 2010). Besides, it was also identified as a potential anticancer agent by inducing apoptosis of cancer cell lines (Yip et al., 2006). Benzethonium was shown to decrease erythrocytes cell size, increase PS exposure, hemolysis and cytosolic Ca^2+^ levels (Lang et al., 2011).

#### None of the Eryptosis Inducers Tested Induce Eryptosis of Naïve and *P. falciparum*-Infected Erythrocytes

Using the eryptosis guidelines we previously developed (4 h incubation period in iRPMI at 4% hematocrit and 37°C), the effect of six eryptosis inducers was measured on naïve and *P. falciparum*-infected erythrocytes. Following incubation with each eryptosis inducer, phenotypic hallmarks of eryptosis were measured, namely PS exposure, cell size, intracellular calcium levels and hemolysis.

**Figure 4:**
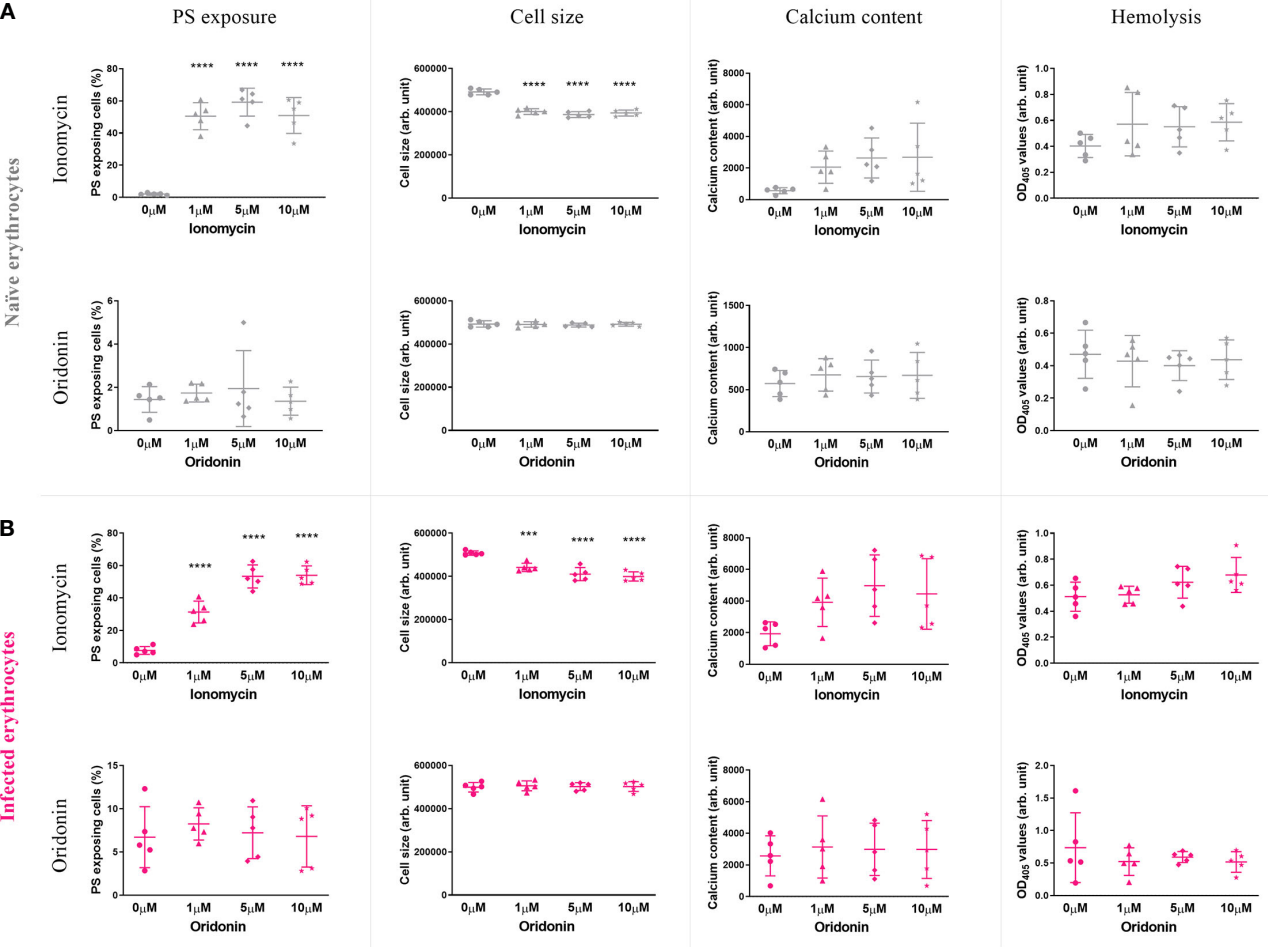
Effect of ionomycin and oridonin on eryptosis hallmarks of naïve **(A)** and *P. falciparum*-infected erythrocytes **(B).** Significant increase of PS exposure and significant decrease of cell size were observed in naïve and infected erythrocytes exposed to 1 µM of ionomycin (4 h in incomplete RPMI). However, no significant increase in these eryptosis hallmarks was observed in naïve and *P. falciparum*-infected erythrocytes exposed up to 10 µM of oridonin. Data presented corresponds to five independent experiments performed in triplicate (N=5). Individual data points represent the means of the three technical replicates for each experiment. The bars represent the mean and SD of the five independent experiments.

The eryptosis measures of naïve and *P. falciparum*-infected erythrocytes exposed to 0, 1, 5, and 10 μM of oridonin, one of the most clinically promising candidates, and the positive control ionomycin (1 μM) are presented in **Figure 4**. As expected, ionomycin induced a significant increase of PS exposure and a decrease in cell size, both in naïve (*p*<0.0001) and in *P. falciparum*-infected erythrocytes (*p*<0.0008), demonstrating the validity of our eryptosis assay. In contrast, oridonin did not induce statistically significant changes in any of the eryptosis markers (PS exposure, cell size, intracellular calcium levels and hemolysis) in naïve or *P. falciparum*-infected erythrocytes.

Next, naïve erythrocytes and *P. falciparum* cultures were also exposed to 0, 1, 5, and 10 μM of benzethonium, amiodarone, BAY 43-9006, cordycepin and apigenin, for 4 h in iRPMI, and the levels of eryptosis hallmarks were measured. Similar to oridonin, none of the other five compounds tested generated multiple eryptosis phenotypic hallmarks on either naïve or infected erythrocytes. Data is summarized in **Table 2** and details are provided in **Supplementary Figures 1**–**5**.

**Table 2:** Eryptosis hallmarks of naïve and *P. falciparum*-infected erythrocytes exposed to seven eryptosis inducers and growth inhibition (IC_50_) on *P. falciparum*.

Compound	Eryptosis hallmarks		*P. falciparum* IC_50_ (μM)
	Naïve erythrocytes	*P. falciparum* – infected erythrocytes	
**Ionomycin**	**↑ PS exposure** **↓ Cell Size** (1 μM)	**↑ PS exposure** **↓ Cell Size** (1 μM)	3.32
**Benzethonium**	Ns	Ns *	0.49
**Oridonin**	Ns	Ns	2.00
**Amiodarone**	**↑** Calcium (5 μM)	Ns	2.10
**BAY 43-9006**	Ns	Ns	7.48
**Cordycepin**	Ns	Ns	12.07
**Apigenin**	Ns	Ns	65.78

Eryptosis hallmarks include PS exposure, cell size, intracellular calcium levels and hemolysis data (see **Figure 4** and **Supplementary Figures 1**–**5** for detailed data on each compound). Ionomycin was used as a positive control. Significant changes (**↑ =** increase or **↓ =** decrease) are indicated with the concentration at which the effect is observed. *: infected erythrocytes incubated with 5 μM benzethonium present an increase in hemolysis levels, which has not been observed at 10 μM.

#### Three Eryptosis Inducers Impact Parasite Viability

**Figure 5:**
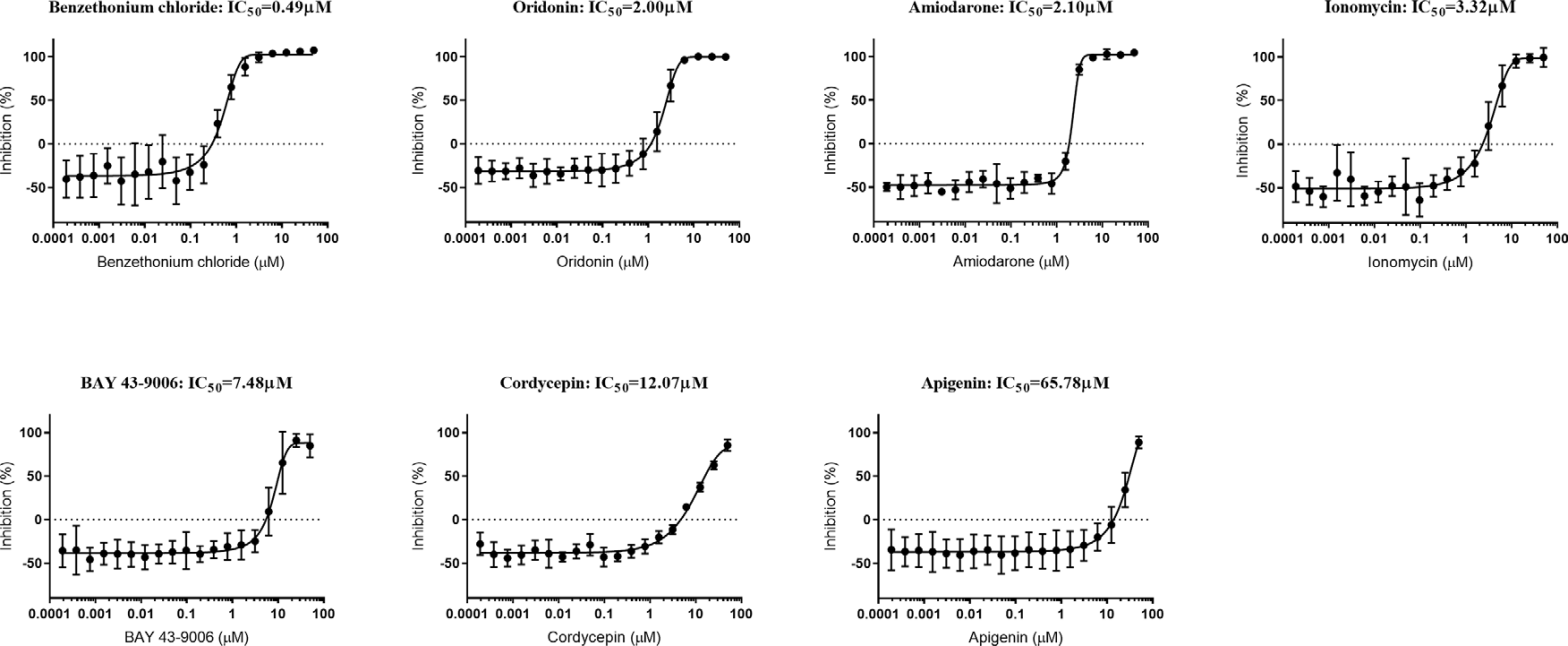
Growth-inhibition curves of *P. falciparum* exposed to seven eryptosis inducers. *In vitro* asynchronous cultures of *P. falciparum* were cultured in cRPMI with a range of concentrations of compounds (up to 50 µM) for 72 h. DNA content was measured as a proxy for parasite growth, and normalized with the positive inhibition control (cultures exposed to chloroquine and artemisinin) and with the negative inhibition control (cultures exposed to DMSO). IC_50_ concentrations are indicated for each compound in µM. Data presented corresponds to five independent experiments performed in triplicate (N=5). Each IC_50_ curve is represented as the mean and SD of the five independent experiments.

Our data demonstrated that none of the six compounds tested have the ability to induce eryptosis of naïve or *P. falciparum*-infected erythrocytes. However, two of these compounds had previously been shown to exhibit an anti-*Plasmodium* activity (Bobbala et al., 2010b; Amiri et al., 2018). Therefore, we tested the ability of the seven eryptosis inducers (including ionomycin) to inhibit *P. falciparum* growth by measuring their IC_50_ values *in vitro* (**Figure 5** and **Table 2**). Cordycepin and apigenin did not impact parasite growth (IC_50_ of 12 and 66 µM, respectively), while five other compounds revealed an IC_50_ below 10 µM. In particular, benzethonium presents a sub-micromolar IC_50_ value of 490 nM, and the IC_50_ of oridonin and amiodarone are ~2 µM. Ionomycin and BAY 43-9006 presented more moderate IC50 values of 3.32 µM and 7.48 µM, respectively. Unlike eryptosis phenotypic assays, parasite growth assays are conducted over 72 h, and therefore require Albumax supplementation to allow parasite survival. The negative growth inhibition control (0.5% (v/v) DMSO) slightly impaired parasite growth (Naidu et al., 2018), more so than the lowest concentrations of drugs tested, therefore at very low drug concentrations the IC_50_ curves exhibit negative values (**Figure 5**). As an additional control, growth inhibition assays were conducted on the antimalaria compounds artemisinin and chloroquine: the IC_50_ values obtained (12 nM and 22 nM, respectively) are comparable to previously published results (Baniecki et al., 2007) (**Supplementary Figure 6**). Collectively, our data strongly indicates that the eryptosis inducers benzethonium, oridonin and amiodarone have antiparasitic effects that are independent of eryptosis induction (**Table 2**).

## Discussion

A variety of eryptosis inducers have been used to study eryptosis of naïve erythrocytes over the past 20 years. More recently, an emerging field of research has tested the potential of eryptosis inducers as antimalarials. In some cases, eryptosis inducers were reported to cause parasite death. However, we have noted that the same studies did not observe death of the non-infected erythrocytes (reviewed in Boulet et al., 2018). Therefore, and despite the observations of parasite death in some cases, we observed major discrepancies in the literature regarding the eryptosis effect of eryptosis inducing compounds. Given that culture conditions differ substantially between eryptosis-focused studies and malaria studies, we hypothesized that these differences underpin the discrepancies observed in the literature, *i.e.* compounds described as eryptosis inducers, did not induce PS exposure of non-infected erythrocytes when tested in malaria culture conditions (reviewed in Boulet et al., 2018). Here, we discuss the contribution of each culture condition variable to eryptosis read-out (Ringer *vs*. RPMI media, hematocrit levels, Albumax supplementation, impact of drug incubation time) and debate the potential of eryptosis inducers as antimalarials.

### Ringer Solution Sensitizes Erythrocytes to Eryptosis

We assessed the impact of the media itself (without eryptosis inducers and Albumax supplementation) on eryptosis and observed that erythrocytes exposed more PS and displayed increased hemolysis when incubated in Ringer when compared to iRPMI (**Figures 2C, D**). This indicates that Ringer solution primes erythrocytes to eryptosis. Further, when erythrocytes were exposed to the strong eryptosis inducer ionomycin (**Figure 3**), the induced hemolysis was stronger in Ringer than in iRPMI. PS exposure was similar in these two media, perhaps due to cells reaching a plateau in PS exposure capacity (as suggested by the time-course analysis, **Figures 3C, D**). Plasmodium culture media is optimized to mimic physiological blood conditions, while Ringer is a nutrient-poor solution. A good example relates to the glucose concentration present in each solution, higher in RPMI (11mM) than in Ringer (5mM). It has been previously shown that glucose depletion enhances eryptosis (Klarl et al., 2006; Bigdelou and Farnoud, 2020), therefore differences in culture media composition could modulate an eryptosis baseline and the nutrient-poor Ringer solution may weaken erythrocytes and pre-dispose them to eryptosis. Interestingly however, when Albumax is added to Ringer solution and RPMI media, no differences in baseline PS exposure or hemolysis are observed (**Figures 2C, D**). This may indicate that addition of Albumax is enough to “rescue” erythrocytes from fragilization by Ringer solution. The contribution of Albumax is discussed in greater details below.

### A Lower Hematocrit Exacerbates Eryptosis Phenotypes

In addition to the difference in media, we investigated the impact of cell density on eryptosis induction. We demonstrated that at 0.4% hematocrit, there is an exacerbated increase in PS exposure and hemolysis induced by BAY 43-9006, when compared to 4% hematocrit (**Figure 1**). This increased susceptibility to BAY 43-9006-induced eryptosis occurring at a lower hematocrit (0.4% *vs*. 4%) may be, at least partly, attributed to the higher amount of drug available per cell: at 0.4% hematocrit, each cell has access to 10-times more BAY 43-9006 molecules than at 4% hematocrit. Additionally, erythrocytes, similar to other cell types, appear to show better viability outcomes with a higher cell density, better reflecting physiological conditions (Ma et al., 2010; Wei et al., 2011).

### Albumax Protects Erythrocytes From Eryptosis

Albumax supplementation (a serum replacement) is required for the continuous growth of *P. falciparum in vitro*. In contrast, eryptosis inducers have previously been tested *in vitro* in the absence of human serum or a synthetic replacement (Lang et al., 2011; Lupescu et al., 2012). Here, we show that addition of Albumax (to Ringer solution or iRPMI media) is sufficient to protect erythrocytes from PS exposure and hemolysis. This is particularly striking when eryptosis is induced following addition of the calcium-ionophore ionomycin where PS exposure remains unchanged for 8 h and hemolysis is not observed (**Figure 3**).

The protein binding effect of Albumax, and its interference with drugs, is well documented (Duffy and Avery, 2017; Charman et al., 2020). Therefore, direct binding of Albumax components to ionomycin and BAY 43-9006 might be partly responsible for the observed death “protection” effect (**Figures 1**, **2**, and **3**). Comparing eryptosis levels in Albumax or in Bovine Serum Albumin (the major contributor of drug-binding in Albumax)-supplemented media would assist in investigating this question further.

Noteworthy, we have demonstrated that Albumax protects cells from eryptosis even in the absence of any drug (**Figures 2C, D**). Given that some of the tested compounds impaired *P. falciparum* growth in the presence of Albumax, we propose that bioavailability cannot solely explain the protective effect of Albumax. Walsh and colleagues suggested that human plasma contains pro-survival factors as it has the capacity to inhibit erythrocytic PS exposure induced by the apoptosis inducer BH3-mimetics (Walsh et al., 2002). Although our study did not use human serum, our results suggest that Albumax might contain pro-survival factors that prevent eryptosis induction. Such pro-survival factors could include Albumax high lipid content. Indeed, certain lipids can protect against cell death and stress, as demonstrated with lipid droplets in nucleated cells (Garcia-Gonzalo and Izpisúa Belmonte, 2008; Petan et al., 2018). Similar to nucleated cells, the presence of lipids in the culture media could improve erythrocyte viability and reduce their susceptibility to cell death induction (Diomede et al., 1993). To identify the Albumax factor(s) responsible for this protective effect against eryptosis, future studies including Albumax fractionation experiments would be required (Gao et al., 2015).

### The Dynamics of PS Exposure Over Time

Although induction of PS exposure in nucleated cells occurs in a matter of minutes (Bevers et al., 1983), eryptosis has predominantly been studied at 48 h post-stimulation. Here we demonstrated that induction of PS exposure in erythrocytes has a rapid onset (of less than 1 h), consistent with observations in other cell types. Moreover, we demonstrated that PS exposure levels increased over time regardless of culture conditions, which corroborates findings suggesting aging erythrocytes are recognized and cleared from circulation through increased PS exposure during senescence (Lang et al., 2011; Lupescu et al., 2012; Zbidah et al., 2012).

### Eryptosis Inducers Do Not Induce Eryptosis of Naïve or *Plasmodium*-Infected Erythrocytes

We selected six eryptosis inducers (amiodarone, BAY 43-9006, cordycepin, oridonin, benzethonium, apigenin) based on their use (or prospective use) in the clinic, their known molecular targets and/or their previously demonstrated antimalarial effects. Using our newly established guidelines to study eryptosis in the context of malaria, we measured the eryptosis effect of these compounds at 1, 5 and 10 μM on naïve and *P. falciparum*-infected erythrocytes. Our study found that none of the six eryptosis inducers presented the ability to induce eryptosis hallmarks, either in naïve or in *P. falciparum*-infected erythrocytes.

Erythrocyte studies have previously reported eryptosis inducing concentrations for oridonin, cordycepin and apigenin on naïve erythrocytes at 25 μM, 31 μM, and 15 μM, respectively (Lui et al., 2007; Jilani et al., 2011; Zbidah et al., 2012). These values are higher than the maximum concentration of 10 μM used in our study and could explain the difference in findings. Drug concentrations above 10 μM might be useful for functional or mechanistic studies, but they are unlikely to be physiological relevant. Similarly, erythrocyte biology studies have previously reported eryptosis inducing concentrations for BAY 43-9006, benzethonium and amiodarone at 1 μM, 5 μM, and 5 μM, respectively (Nicolay et al., 2007; Lang et al., 2011; Lupescu et al., 2012). Our study did not detect any eryptosis inducing effect for these compounds up to a concentration of 10 μM. We attribute the differences between previously published studies and our findings to differences in the experimental conditions, as discussed above.

Among the six eryptosis inducer compounds tested in our study, only apigenin and amiodarone have been previously tested on malaria parasites (Nicolay et al., 2007; Fallatah and Georges, 2017; Amiri et al., 2018). In these studies, the effect of apigenin on eryptosis of infected cells was not measured, so we cannot compare our findings with previous data (Fallatah and Georges, 2017; Amiri et al., 2018). On the other hand, amiodarone was shown to increase PS exposure of *P. falciparum*-infected erythrocytes at 10 μM (Bobbala et al., 2010b), contrasting with our own findings. This discrepancy could be due to the different parasite strains used (BinH *vs*. 3D7), the lower hematocrit (2% *vs*. 4%), the longer incubation time (24 h *vs*. 4 h) and/or the synchronicity of parasites (synchronized late stages *vs*. asynchronous) (Bobbala et al., 2010b).

### Benzethonium, Amiodarone, and Oridonin Impair *Plasmodium* Growth *via* an Eryptosis-Independent Mechanism

Among the eryptosis inducer compounds tested, our study identified that three compounds (benzethonium, oridonin and amiodarone) display low *P. falciparum* IC_50_ values (≤2 μM). Ionomycin and BAY 43-9006 display moderate IC_50_ (<10 μM), while cordycepin and apigenin IC_50_ values measured above 10 μM.

An antiparasitic activity of apigenin has been previously reported, both against *P. berghei* in mice and *P. falciparum in vitro* (Fallatah and Georges, 2017; Amiri et al., 2018). Our study measured a higher IC_50_ value than the one previously published (65.78 μM *vs*. 36.02 μM) (Fallatah and Georges, 2017), a difference that can be attributed to differences within the growth inhibition assays. Nevertheless, the high IC_50_ values would preclude apigenin from been considered an attractive antimalarial candidate.

Benzethonium displayed the lowest IC_50_ value (0.49 μM) and could therefore be considered a promising antimalarial. However, its toxicity effects will need to be abrogated before being investigated further as a potential antimalarial (National Center for Biotechnology Information; Sreevidya et al., 2018). Benzethonium is used as an antimicrobial, acting by disrupting lipid bilayers, and has been identified as an anti-cancer compound by inducing apoptosis (Yip et al., 2006). However, no consistent significant increase in hemolysis was observed both in naïve and *P. falciparum*-infected erythrocytes, suggesting that membrane disruption is not the mode of action. It is therefore possible that benzethonium targets a parasite-specific pathway.

Amiodarone, a clinically used antiarrhythmic drug, inhibited *P. falciparum* growth with an IC_50_ of 2.10 μM. This antiparasitic effect is in agreement with a study describing reduced parasitemia of *P. falciparum in vitro* and *P. berghei in vivo* (Bobbala et al., 2010b). Interestingly, a typical 400mg dose of amiodarone in adults leads to a peak plasma concentration in the same order of magnitude as the IC_50_ value we measured here (Goldschlager et al., 2000). However, amiodarone can cause serious side effects, and is only recommended in case of life-threatening arrythmia; more work is therefore required before it can be proposed as a novel antimalarial candidate. As an antiarrhythmic agent, amiodarone acts by inhibiting calcium, potassium and sodium channels. Inhibition of ion channels on either the erythrocyte surface or the parasite interface could be part of the mechanism of action.

Similarly, oridonin displayed a low IC_50_ (2 μM). One oridonin derivative (HAO472) is currently in phase I clinical trials in China for the treatment of leukemia (Ding et al., 2016). As such, this compound might be the most promising antimalarial candidate tested in this study. In cancer cells lines, oridonin and oridonin-like compounds were shown to regulate many pathways, including NF-κB, ROS, p53/p21, to inhibit proliferation and induce apoptosis (Ding et al., 2016). At this stage, it is therefore difficult to infer the mechanism of action behind oridonin antimalarial activity.

### PS Exposure During a Malaria Infection: Turning the Tables on *Plasmodium*

PS exposure at the surface of infected and non-infected erythrocytes during a malaria infection could be detrimental to the host. PS exposure by infected cells has been linked with an increase in their cytoadhesion and sequestration, both in *P. falciparum* (Zhang et al., 2018) and in *P. vivax* malaria (Totino and Lopes, 2017). Further, PS exposure of uninfected erythrocytes and increased levels of anti-PS auto-antibodies have been proposed as key mechanisms leading to the loss of non-infected erythrocytes, therefore contributing to malarial anemia (Jakeman et al., 1999; Totino et al., 2010; Barber et al., 2019). However, compared to their non-infected counterparts, a larger proportion of infected erythrocytes expose PS (Schwartz et al., 1987). This propensity of infected erythrocytes to expose PS could be used to target antimalarials specifically to infected cells using peptides that accumulate predominantly in PS-exposing cells (Lawrence et al., 2018) or with liposomes conjugated to PS binding peptides (Tagami et al., 2015). These techniques offer promising alternatives for antimalarial development that could be explored in the future.

### Conclusions and Future Directions

We have shown that experimental culture conditions previously used to study eryptosis weaken erythrocytes and predispose them to eryptosis. Media supplementation with Albumax appears to strongly protect against eryptosis, even in the presence of potent eryptosis inducers. In this study, we have developed and implemented novel experimental guidelines to allow the study of eryptosis. Using these guidelines, we have shown that compounds previously identified as eryptosis inducers failed to induce eryptosis of naïve and *P. falciparum*-infected erythrocytes *in vitro*. Interestingly, three of the compounds tested in our study present a promising antiparasitic activity. In contrast to previous reports, the effect of these compounds appears to be independent of eryptosis, although their exact mode of action remains to be elucidated.

## Data Availability Statement

The original contributions presented in the study are included in the article/**Supplementary Material**. Further inquiries can be directed to the corresponding author.

## Ethics Statement

Use of human erythrocytes was approved by the La Trobe University Research Ethics Committee (ethics number HEC17‐013) and an Australian Red Cross Blood Service Agreement (Deed 19-05VIC76 01).

## Author Contributions

CB, TG, and TC planned the experimental work and analyzed the data. CB and TG performed the experimental work. CB and TC wrote the manuscript. All authors contributed to the article and approved the submitted version.

## Funding

Funding support was provided to TC by La Trobe University Start up Grant (2018–2021), Infrastructure grants (2017, 2019, 2020), and Research Focus Area grant (2018–2020). CB received a Postgrad Research scholarship from La Trobe University.

## Conflict of Interest

The authors declare that the research was conducted in the absence of any commercial or financial relationships that could be construed as a potential conflict of interest.

## Supplementary Material

Supplementary Figure 1 Effect of benzethonium on eryptosis levels of naïve and *P. falciparum*-infected erythrocytes. Naïve erythrocytes or *P. falciparum* cultures were incubated in incomplete RPMI for 4h in presence of 0, 1, 5 or 10μM benzethonium. PS exposure, cell size, calcium levels were measured by flow cytometry, and hemolysis was measured by absorbance assay. N=5, except for hemolysis where n=4. Mean and SD.

Supplementary Figure 2 Effect of amiodarone on eryptosis levels of naïve and *P. falciparum*-infected erythrocytes. Naïve erythrocytes or *P. falciparum* cultures were incubated in incomplete RPMI for 4h in presence of 0, 1, 5 or 10μM amiodarone. PS exposure, cell size, calcium levels were measured by flow cytometry, and hemolysis was measured by absorbance assay. N=6 naïve, except for hemolysis where n=5. Mean and SD.

Supplementary Figure 3 Effect of BAY 43-9006 on eryptosis levels of naïve and *P. falciparum*-infected erythrocytes. Naïve erythrocytes or *P. falciparum* cultures were incubated in incomplete RPMI for 4h in presence of 0, 1 or 10μM BAY 43-9006. PS exposure, cell size and calcium levels were measured by flow cytometry. N=5. Mean and SD.

Supplementary Figure 4 Effect of cordycepin on eryptosis levels of naïve and *P. falciparum*-infected erythrocytes. Naïve erythrocytes or *P. falciparum* cultures were incubated in incomplete RPMI for 4h in presence of 0, 1, 5 or 10μM cordycepin. PS exposure, cell size, calcium levels were measured by flow cytometry, and hemolysis was measured by absorbance assay. N=5. Mean and SD.

Supplementary Figure 5 Effect of apigenin on eryptosis levels of naïve and *P. falciparum*-infected erythrocytes. Naïve erythrocytes or *P. falciparum* cultures were incubated in incomplete RPMI for 4h in presence of 0, 1, 5 or 10μM apigenin. PS exposure, cell size, calcium levels were measured by flow cytometry, and hemolysis was measured by absorbance assay. N=6 for naïve and N=5 for infected erythrocytes. Mean and SD.

Supplementary Figure 6 Growth-inhibition curves of *P. falciparum* exposed to artemisinin and to chloroquine. In vitro asynchronous cultures of *P. falciparum* were cultured in cRPMI with a range of concentrations of compounds (up to 50μM) for 72h. DNA content was measured as a proxy for parasite growth, and normalised with the positive inhibition control (cultures exposed to chloroquine and artemisinin) and with the negative inhibition control (cultures exposed to DMSO). IC_50_ concentrations are indicated for each compound in μM. Data presented corresponds to five independent experiments performed in triplicate (N=5 for artemisinin, N=4 for chloroquine). Each IC_50_ curve is represented as the mean and SD of the independent experiments.
