# Supplementary figures and images for: Eryptosis and Malaria: New Experimental Guidelines and Re-Evaluation of the Antimalarial Potential of Eryptosis Inducers
Source: Front Cell Infect Microbiol. 2021 Mar 12;11:630812. doi: 10.3389/fcimb.2021.630812 (PMC7994622; doi:10.3389/fcimb.2021.630812)

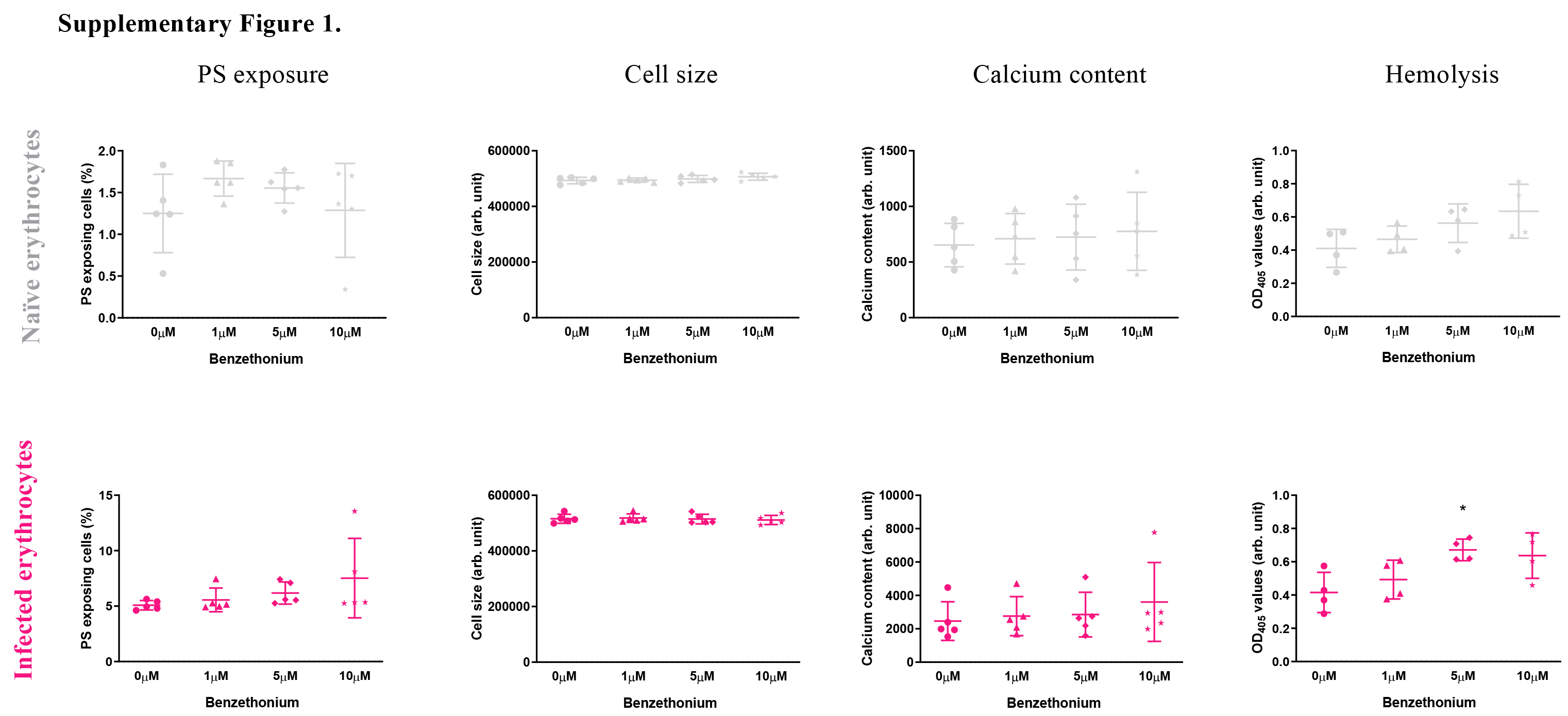

Supplement: Supplementary Figure 1 — Effect of benzethonium on eryptosis levels of naïve and P. falciparum-infected erythrocytes. Naïve erythrocytes or P. falciparum cultures were incubated in incomplete RPMI for 4h in presence of 0, 1, 5 or 10μM benzethonium. PS exposure, cell size, calcium levels were measured by flow cytometry, and hemolysis was measured by absorbance assay. N=5, except for hemolysis where n=4. Mean and SD. [file Image_1.tif]

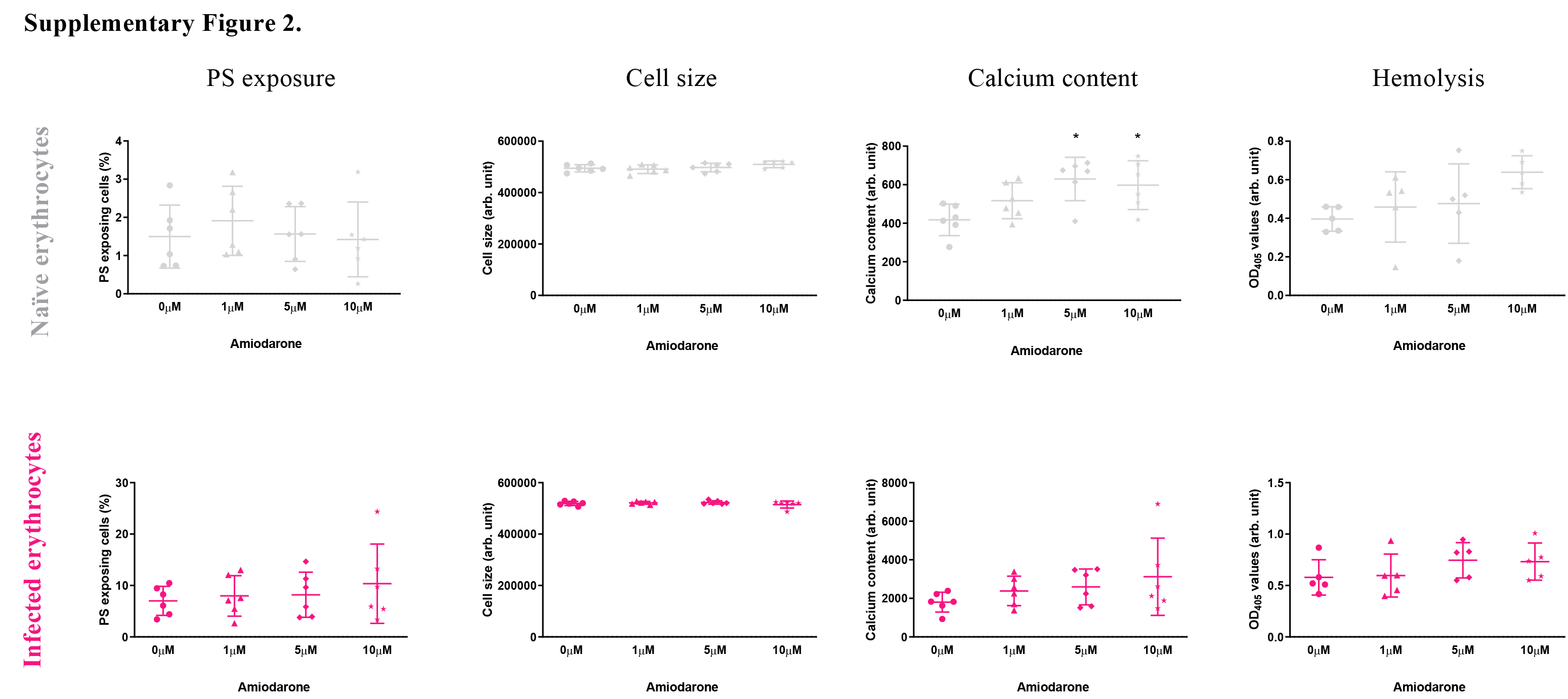

Supplement: Supplementary Figure 2 — Effect of amiodarone on eryptosis levels of naïve and P. falciparum-infected erythrocytes. Naïve erythrocytes or P. falciparum cultures were incubated in incomplete RPMI for 4h in presence of 0, 1, 5 or 10μM amiodarone. PS exposure, cell size, calcium levels were measured by flow cytometry, and hemolysis was measured by absorbance assay. N=6 naïve, except for hemolysis where n=5. Mean and SD. [file Image_2.tif]

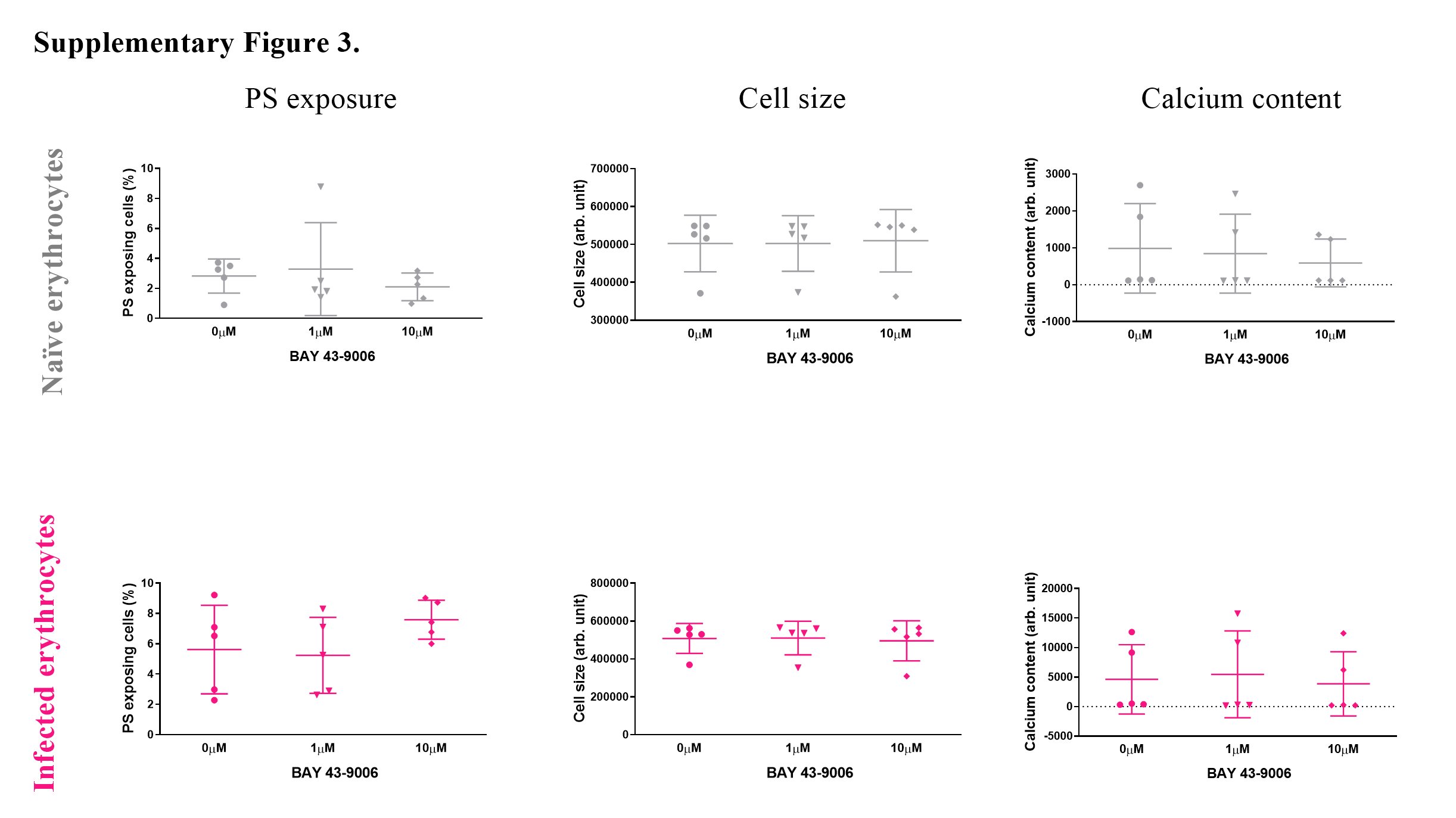

Supplement: Supplementary Figure 3 — Effect of BAY 43-9006 on eryptosis levels of naïve and P. falciparum-infected erythrocytes. Naïve erythrocytes or P. falciparum cultures were incubated in incomplete RPMI for 4h in presence of 0, 1 or 10μM BAY 43-9006. PS exposure, cell size and calcium levels were measured by flow cytometry. N=5. Mean and SD. [file Image_3.tiff]

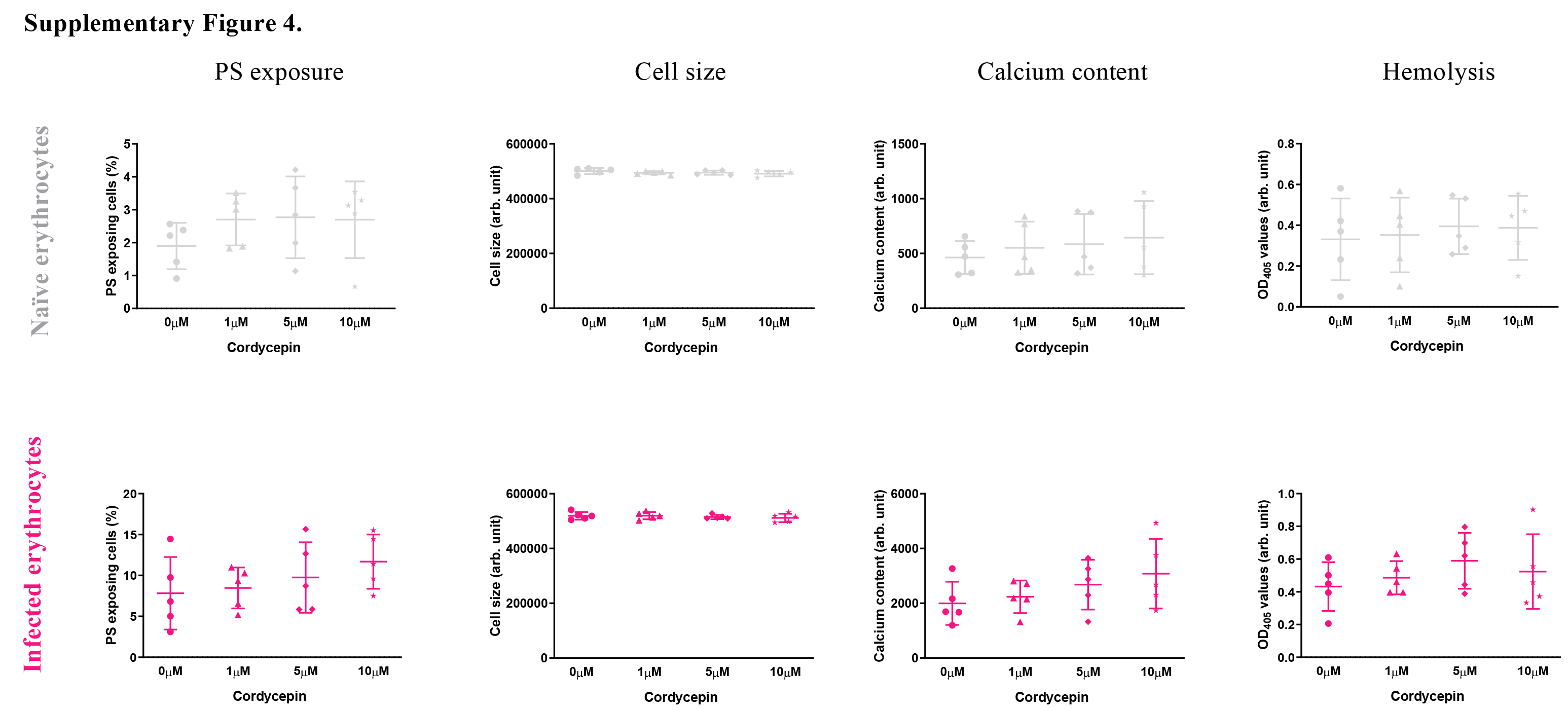

Supplement: Supplementary Figure 4 — Effect of cordycepin on eryptosis levels of naïve and P. falciparum-infected erythrocytes. Naïve erythrocytes or P. falciparum cultures were incubated in incomplete RPMI for 4h in presence of 0, 1, 5 or 10μM cordycepin. PS exposure, cell size, calcium levels were measured by flow cytometry, and hemolysis was measured by absorbance assay. N=5. Mean and SD. [file Image_4.tif]

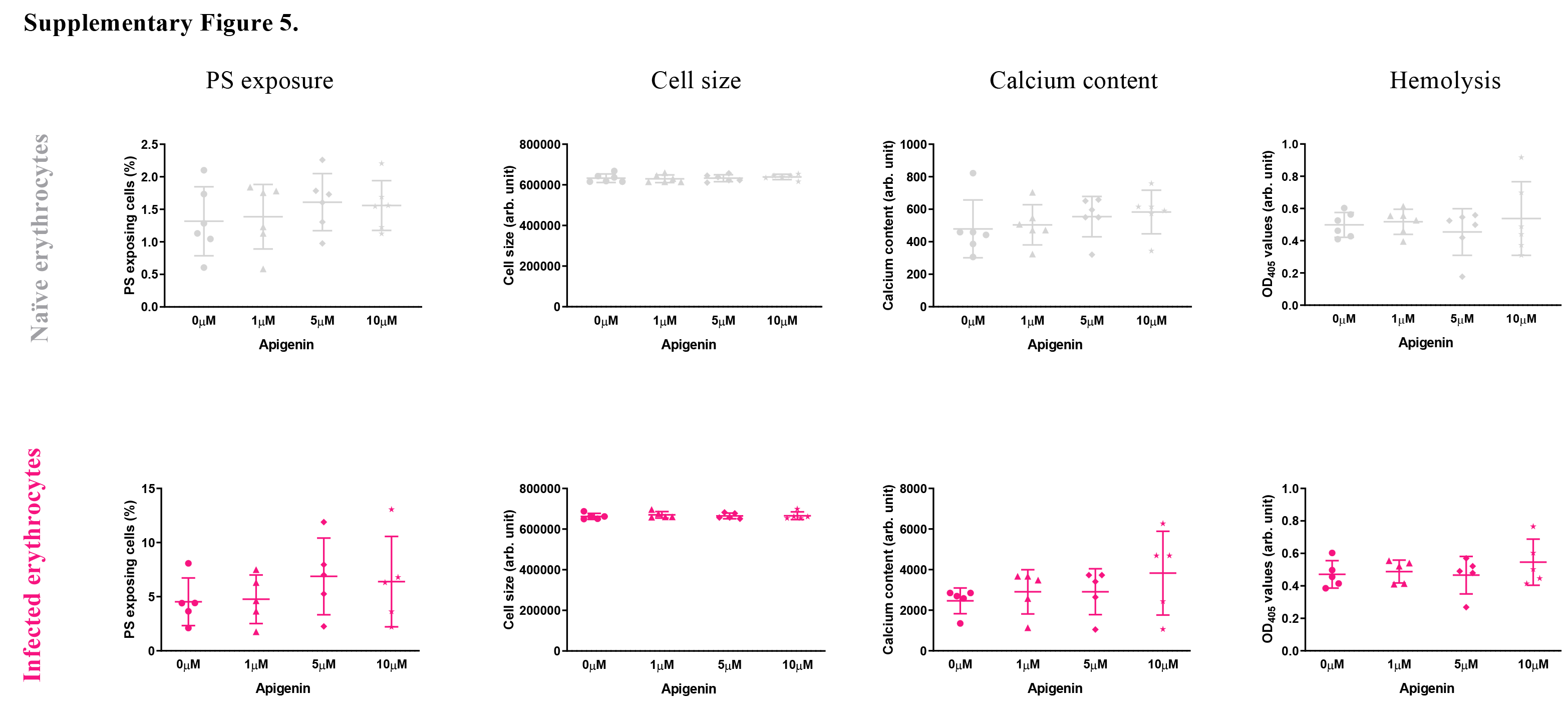

Supplement: Supplementary Figure 5 — Effect of apigenin on eryptosis levels of naïve and P. falciparum-infected erythrocytes. Naïve erythrocytes or P. falciparum cultures were incubated in incomplete RPMI for 4h in presence of 0, 1, 5 or 10μM apigenin. PS exposure, cell size, calcium levels were measured by flow cytometry, and hemolysis was measured by absorbance assay. N=6 for naïve and N=5 for infected erythrocytes. Mean and SD. [file Image_5.tif]

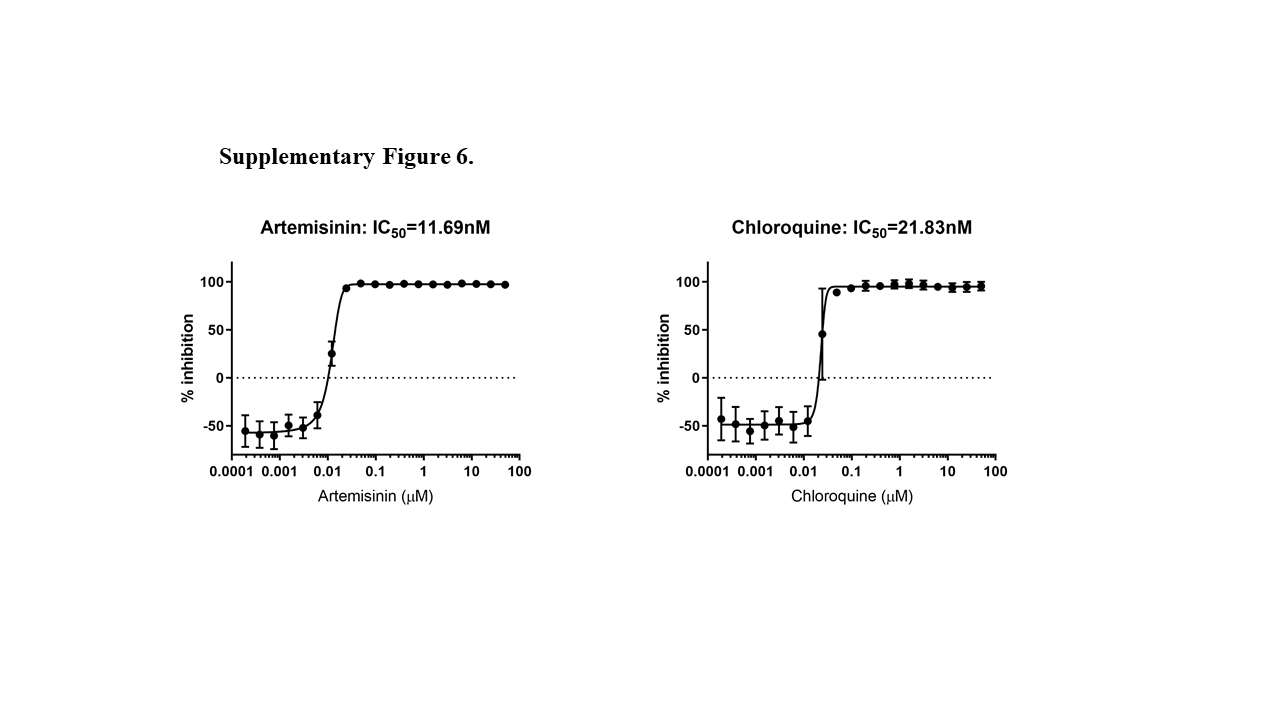

Supplement: Supplementary Figure 6 — Growth-inhibition curves of P. falciparum exposed to artemisinin and to chloroquine. In vitro asynchronous cultures of P. falciparum were cultured in cRPMI with a range of concentrations of compounds (up to 50μM) for 72h. DNA content was measured as a proxy for parasite growth, and normalised with the positive inhibition control (cultures exposed to chloroquine and artemisinin) and with the negative inhibition control (cultures exposed to DMSO). IC50 concentrations are indicated for each compound in μM. Data presented corresponds to five independent experiments performed in triplicate (N=5 for artemisinin, N=4 for chloroquine). Each IC50 curve is represented as the mean and SD of the independent experiments. [file Image_6.tif]
